# Genome-wide identification, characterization and classification of ionotropic glutamate receptor genes (iGluRs) in the malaria vector *Anopheles sinensis* (Diptera: Culicidae)

**DOI:** 10.1186/s13071-017-2610-x

**Published:** 2018-01-15

**Authors:** Ting-Ting Wang, Feng-Ling Si, Zheng-Bo He, Bin Chen

**Affiliations:** Chongqing Key Laboratory of Vector Insects; Institute of Entomology and Molecular Biology, Chongqing Normal University, Chongqing, People’s Republic of China

**Keywords:** *Anopheles sinensis*, iGluRs, Genome-wide identification, Characterization, Classification

## Abstract

**Background:**

Ionotropic glutamate receptors (iGluRs) are conserved ligand-gated ion channel receptors, and ionotropic receptors (IRs) were revealed as a new family of iGluRs. Their subdivision was unsettled, and their characteristics are little known. *Anopheles sinensis* is a major malaria vector in eastern Asia, and its genome was recently well sequenced and annotated.

**Methods:**

We identified iGluR genes in the *An. sinensis* genome, analyzed their characteristics including gene structure, genome distribution, domains and specific sites by bioinformatic methods, and deduced phylogenetic relationships of all iGluRs in *An. sinensis*, *Anopheles gambiae* and *Drosophila melanogaster*. Based on the characteristics and phylogenetics, we generated the classification of iGluRs, and comparatively analyzed the intron number and selective pressure of three iGluRs subdivisions, iGluR group, Antenna IR and Divergent IR subfamily.

**Results:**

A total of 56 iGluR genes were identified and named in the whole-genome of *An. sinensis*. These genes were located on 18 scaffolds, and 31 of them (29 being IRs) are distributed into 10 clusters that are suggested to form mainly from recent gene duplication. These iGluRs can be divided into four groups: NMDA, non-NMDA, Antenna IR and Divergent IR based on feature comparison and phylogenetic analysis. IR8a and IR25a were suggested to be monophyletic, named as Putative in the study, and moved from the Antenna subfamily in the IR family to the non-NMDA group as a sister of traditional non-NMDA. The generated iGluRs of genes (including NMDA and regenerated non-NMDA) are relatively conserved, and have a more complicated gene structure, smaller ω values and some specific functional sites. The iGluR genes in *An. sinensis*, *An. gambiae* and *D. melanogaster* have amino-terminal domain (ATD), ligand binding domain (LBD) and Lig_Chan domains, except for IR8a that only has the LBD and Lig_Chan domains. However, the new concept IR family of genes (including regenerated Antenna IR, and Divergent IR), especially for Divergent IR are more variable, have a simpler gene structure (intron loss phenomenon) and larger ω values, and lack specific functional sites. These IR genes have no other domains except for Antenna IRs that only have the Lig_Chan domain.

**Conclusions:**

This study provides a comprehensive information framework for iGluR genes in *An. sinensis*, and generated the classification of iGluRs by feature and bioinformatics analyses. The work lays the foundation for further functional study of these genes.

**Electronic supplementary material:**

The online version of this article (10.1186/s13071-017-2610-x) contains supplementary material, which is available to authorized users.

## Background

Sensing the environmental small molecules is a kind of important ability for all species in bacteria, plants and animals. Diverse rapid and specific communication between cells and between individuals principally relies on chemical signals. Insects are able to sense a diversity of environmental chemicals such as bitter, sweet, salty tastants, odors, humidity, pheromones and carbonated water [1, 2]. External molecular cues are usually recognized by detecting receptor proteins on cell surface, which can trigger a series of changes like gene expression, physiology or behavior both in cells and organisms. In the nervous system, intercellular communication occurs between neurons at synapses. To respond action potentials, cell terminal presynaptic membranes release diffusible neurotransmitters, and these neurotransmitters are recognized by receptor proteins in postsynaptic cell membranes, which thus can induce neuronal depolarization and continued propagation of action potentials.

One of the best characterized synaptic communication mechanism is mediated by ionotropic glutamate receptors (iGluRs) and neurotransmitter glutamate [3]. iGluR is a kind of conserved ligand-gated ion channels genes, which have been shown to be involved in mediating fast neuronal responses in excitatory synapses via combining the neurotransmitter glutamate [4], representing an important signaling mechanism by which cells can modify their internal physiology in response to external chemical signals [5]. iGluRs are pharmacologically and molecularly classified into three major classes [6] across vertebrates and invertebrates, such as humans, *Mus musculus* [7], *Drosophila melanogaster* [8], *Caenorhabditis elegans* [9] and *Aplysia californica* [10]. These three classes of iGluRs are AMPA (α-amino-3-hydroxy-5-methyl-4-isoxazole propionate) receptors, Kainate receptors and NMDA (N-methyl-D-aspartate) receptors, respectively. Generally, the AMPA and Kainate receptors are further grouped together as non-NMDA type. AMPA receptors mediate the vast majority of fast excitatory synaptic transmission in the vertebrate brain, while Kainate receptors have a subtler modulatory role in this process. NMDA receptors require two agonists, glutamate and glycine for activation, and function in synapsis and neuronal plasticity.

Recently, a new family of ionotropic glutamate receptors, the ionotropic receptors (IRs) family was revealed as a new class of chemosensory receptors in *Drosophila melanogaster* [11], and they can detect a variety of volatile chemical molecules and chemical signal between cells. Traditional iGluRs and IRs have been identified and characteristic in many insects, mollusc and vertebrate [7]. In *D. melanogaster* and *Anopheles gambiae*, there are 12, 9 iGluRs and 66, 46 IRs, respectively. Like iGluRs, the moleculars of IRs are characteristic of having a bipartite ligand-binding domain (LBD) with two half-domains S1 and S2, a Lig_Chan domain that is formed by three transmembrane segments (M1, M2, M3) and a ion channel pore (P) [12]. But IRs lack extracellur amino-terminal domain (ATD) that is characteristic of traditional iGluRs (except for IR8a and IR25a), and the LBD of IRs lack one or more characteristic residues (ligand-binding sites) that directly contact glutamate ligand found in iGluRs [7].

In a previous study [7], the IR family was classified into two different subfamilies: Antenna IR subfamily and Divergent IR subfamily based on the tissues of their expression and phylogenetic relationship. The Antenna IRs subfamily genes are conserved and expressed in antennae, and were suggested to be novel olfactory receptors in insects. But the Divergent IRs are divergent in sequence homology and express in gustatory organs or other tissues of insects [7]. *IR8a* and *IR25a* are classified into Antenna IR subfamily, but they show some specificities. First, IR8a and IR25a of *D. melanogaster* also have ATD that is characteristic of traditional iGluRs and can combine accessory factors. Secondly, IR8a and IR25a have conserved ligand-binding domain (LBD), and 3 and 2 ligand-binding sites in the LBD, respectively [11]. Moreover, the phylogenetic relationships showed that IR8a and IR25a cluster with traditional iGluRs rather than with IRs [13, 14]. Therefore, the classification for traditional iGluRs and IRs has not been well settled so far.

*Anopheles sinensis* (Diptera, Culicidae) is one of the major malaria vector mosquitoes in China and southeastern Asia with wide distribution from Afghanistan to northern China, Korea, Japan, Taiwan, and southward into western Indonesia [15, 16]. The iGluR genes of *An. sinensis* genome have not been comprehensively analyzed. In this study, we identified and classified the candidate iGluR genes in of *An. sinensis* whole-genome, and conducted a series of bioinformatics analyses on their characteristics, including the structure, genome distribution, selective pressure and phylogenetic relationships of these genes, and the domains, motifs and specific functional sites of their amino acid sequences. As a result, we established a novel classification criterion, moved IR8a and IR25a into non-NMDA receptors (traditional iGluRs) from IRs based on their similar specific sites and phylogenetic relationship. This study established an information framework of *An. sinensis* iGluR genes, and enriched gene data of traditional iGluRs and IRs, which are beneficial for further research on gene expression, regulation, signal transduction, etc. of these genes.

## Methods

### Genome and transcriptome sequence sources

The genome and transcriptome of *Anopheles sinensis* were sequenced using Illumina HiSeq™ 2000 (California, USA) according to the manufacturer’s instructions in Beijing Genomics Institute (BGI, Shenzhen, China and assembled and annotated by the Institute of Entomology and Molecular Biology, Chongqing Normal University, China. The former has been preparing for publication in the institute, and the later was published and downloaded from the NCBI GenBank database (https://www.ncbi.nlm.nih.gov/) as an EST database [15]. An additional set of transcriptome sequences of *An. sinensis*, published in Nanjing Medical University [17], was also retrieved from GenBank and used in the present study. The amino acid sequences of traditional iGluRs and IRs of *An. gambiae*, *Aedes aegypti*, *Culex quinquefasciatus* and *D. melanogaster* were retrieved from VectorBase (https://www.vectorbase.org/) and NCBI GenBank database.

### Genome-wide identification of candidate iGluRs in *An. sinensis*

Three procedures were used for the genome-wide identification of candidate iGluRs in *An. sinensis*. First, we used the iGluRs of *An. gambiae*, *Ae. aegypti*, *Cx. quinquefasciatus* and *D. melanogaster* as query sequences to perform TBLASTn and BLASTP homology searches against *An. sinensis* genome database and amino acid database, with threshold value at E-value < 1 × 10^−5^, respectively. Secondly, HMM file, Lig_Chan (Pfam 00060).hmm, representing the Hidden Markov Model (HMM) of iGluRs was downloaded from Pfam (v.27.0) [18], and used to search against the amino acid database of *An. sinensis* using the HMMER3 software program [19]. Thirdly, additional iterative BLAST searches against genome and amino acid database of *An. sinensis* were conducted using sequences obtained in the earlier two procedures until no new sequences were encountered. The software Fgenesh^+^ (http://www.softberry.com) was used to predict the candidate iGluR genes with the genome sequences extracted, and the candidate protein-coding sequences of iGluR genes were then translated to amino acid sequences. The amino acid sequences translated and obtained in BLAST searches were combined, and the redundant sequences were eliminated. The candidate sequences of iGluRs after the elimination of redundancy were subjected to search against the Conserved Domain Database (CDD) (https://www.ncbi.nlm.nih.gov/cdd/) and Vectorbase to detect the domains and the homologous genes in *An. gambiae*, respectively, and confirm the whole-genome identification.

### Characterization analysis of iGluRs in *An. sinensis*

Compute pI/Mw (http://web.expasy.org/compute_pi/) and SignalP 4.1 (http://www.cbs.dtu.dk/services/SignalP/) were used to predict the theoretical molecular mass and isoelectric point, and signal peptide of aa sequences of all iGluR genes investigated, respectively. The online TargetP (http://www.cbs.dtu.dk/services/TargetP/) was used for cellular location of these iGluR aa sequences. The exon-intron gene structures of *An. sinensis* iGluR genes were predicted through mapping of the corresponding genomic and transcript sequences, with the aid of a Hidden Markov Model-based gene structure predictor (www.Softberry.com), and displayed with the online software Gene Structure Display Sever (http://gsds.cbi.pku.edu.cn/).

Interproscan (http://www.ebi.ac.uk/interpro/search/sequence-search) and NCBI Blast were used to predict conserved domains and specific functional sites, and the online web MEME (http://alternate.meme-suite.org/tools/meme) was used to predict conserved motifs. ClustalW [20] was used to conduct multiple sequence alignments, and the GeneDoc program [21] was used for the examination of their conservation and mark of domains, motifs and ligand-binding sites. All iGluR genes investigated were divided into three groups based on their characteristics: (i) iGluR group including NMDA and non-NMDA (Kainate, AMPA, Putative); (ii) representative Antenna IR subfamily; and (iii) representative Divergent IR subfamily.

### Phylogenetic analysis of iGluRs in *An. sinensis*, *An. gambiae* and *D. melanogaster*

The amino acid sequences of traditional iGluRs and IRs predicted in *An. sinensis*, along with the iGluRs and IRs of *An. gambiae* [22] and *D. melanogaster* [11] were aligned using ClustalW [23]. The residue rows with low-quality alignment were removed manually to obtain final high-quality alignments of 150–350 residues, and then the best model of substitution, WAG model to infer the phylogeny was selected using “find best model” in Mega 5.0 [23]. The phylogenetic tree of these iGluRs and IRs was constructed with Mega 5.0 using the maximum likelihood (ML) method under the WAG model, with bootstrap resampling of 1000 replicates. The ML tree was then viewed and graphically edited with iTol (http://itol.embl.de/) [24]. The bootstrap values larger than 50% were marked on the nodes of the phylogenetic tree to discuss the phylogenetic relationship of NMDA, non-NMDA, Antenna IR subfamily and Divergent IR subfamily.

### Calculation of d_N_/d_S_ (ω) value of iGluR genes between *An. sinensis* and *An. gambiae*

The values of nonsynonymous substitution ratios (d_N_), synonymous substitution ratios (d_S_) and d_N_/d_S_ (ω) are all important parameters to judge selective force and conservation of genes. In this study, we inferred the d_N_/d_S_ ratio (ω) by maximum likelihood as implemented in PAML [25]. The MUSCLE codon in Mega 5.0 was used to conduct pairwise sequence alignment for coding regions of orthologous iGluR genes between *An. sinensis* and *An. gambiae*, the terminators of these genes were manually deleted, and the alignment was saved as FASTA format file. The FASTA format file was transformed to PAML format file, and then put it to yn00 of PAML software to calculate the value of d_N_, d_S_ and d_N_/d_S_ (ω) of iGluR genes in *An. sinensis* under M0 model. All PAML analyses were run three times using different input parameters to avoid local optima. Pseudogenes and incomplete genes were avoided in these analyses, and residue columns with gaps were omitted in the d_N_/d_S_ calculations.

## Results and discussion

### Identification and nomenclature of iGluRs in *An. sinensis* genome

We identified 56 putative iGluR genes in the *An. sinensis* genome, all of which had full-length protein-coding sequences except for 5 genes (*AsGluRIIb1, AsGluRIIb2*, *AsIR64a.1*, *AsIR64a.2* and *AsIR75k.1*). In the 56 genes, 16 were supported by transcripts, although 38 genes did not have transcript support but their amino acid sequences were characteristic of functional domains and motifs (see the following section), and shared high sequence identity (> 30%) with reported insect iGluRs (Additional file 1: Table S1, Additional file 2: Table S2). These 38 genes are mostly IR genes, and only express in antennae or were not found to express in any tissues in *Drosophila* [11]. The transcription data used in the present study were produced through RNA-seq, and based on the pooled samples of *An. sinensis* adult females and males at the age of the third day post-emergence. The expression quantity of these 38 genes might be lower than the detection threshold in the RNA-seq analysis. Therefore, these 54 genes were considered to be functional genes. The remaining 2 iGluR genes (*AsIR100h* and *AsIR101*), although having full-length protein-coding sequences, neither had any domain, motif nor transcript support, and thus they were treated as possible pseudogenes.

The iGluR number (56 genes, including two pseudogenes) in *An. sinensis* was comparable with that in *An. gambiae* (55 genes, also including 2 pseudogenes) [22]. Thirty-nine iGluR genes showed to be 1:1 orthologous between these two species with the identity of amino acid (aa) sequence larger than 30%. Eight genes in *An. gambiae* (*AgGluRIIc*, *AgGluRIIe*, *AgGluRI*, *AgIR100a*, *AgIR100i*, *AgIR134 AgIR139* and *AgIR142*) were lacking in the *An. sinensis* genome, but four in *An. gambiae* (*AgGluRIIb*, *AgIR75d*, *AgIR75k* and *AgIR64a*) had 2, 3, 2 and 2 orthologous genes in *An. sinensis*, respectively, and one in *An. sinensis* (*AsIR143*) had no homologue in *An. gambiae*. In addition, *IR41t* gene had one and two duplications in *An. sinensis* (*AsIR41t.2*) and *An. gambiae* (*AgIR41t.1* and *AgIR41t.2*), respectively, and IR140 had six and two duplications in *An. sinensis* (*AsIR140.1*-*AsIR140.6*) and *An. gambiae* (*AgIR140.1* and *AgIR140.2*), respectively (Additional file 2: Table S2).

The iGluR gene numbers in *An. sinensis* and *An. gambiae* were obviously less than those of two Culicinae mosquito species earlier reported (91 and 71 iGluR genes in *Ae. aegypti* and *Cx. quinquefasciatus*, respectively) [7]. iGluRs of mosquitoes mainly function for reception of environmental chemical signals, such as bitter, sweet, salty tastants, pheromones, odors etc. [1, 2], and the obvious gene number difference might be due to their different life habits. *Anopheles sinensis* and *An. gambiae*, belonging to the subfamily Anophelinae, are nocturnal with indoor ingestion and oviposition, whereas *Ae. aegypti* and *Cx. quinquefasciatus*, belonging to the subfamily Culicinae, are diurnal with outdoor ingestion and oviposition [26, 27].

All *An. sinensis* iGluR genes were named based on the corresponding iGluRs’ names of *An. gambiae*, specific sites, domains and phylogenetic relationship (described afterwards), and standardized iGluRs nomenclature system [7, 28]. *Anopheles sinensis* iGluR names are preceded by a two-letter species abbreviation (*Anopheles sinensis* = *As.*). The iGluR genes with 1:1 *An. gambiae* orthologues were given the same name as those in *An. gambiae* (e.g. *AsIR25a*), and if there were multiple copies for a single *An. gambiae* homologous gene, their copies were given the same name followed by a point and a number (e.g. *AsIR75k.1*, *AsIR75k.2*).

### Characteristics, structure and location of *An. sinensis* iGluR genes

The *An. sinensis* 49 iGluR genes with complete amino acid (aa) sequences encode 333–1178 aa, 31 of which (63%) encode 500–700 aa. All of these 31 aa sequences have similar domain and motif structures although each of their sequences are different. The 49 iGluRs all have the theoretical molecular weight from 38.63 kD to 183.42 kD, and the isoelectric point (IP) ranging from 5.02 to 9.79, and 26 of them have signal peptide. All of these 49 iGluRs were located on cell membrane, which correspond to their function as an ion channel. The detailed information of biochemical properties of these *An. sinensis* iGluR are listed in Additional file 1: Table S1.

**Fig. 1:**
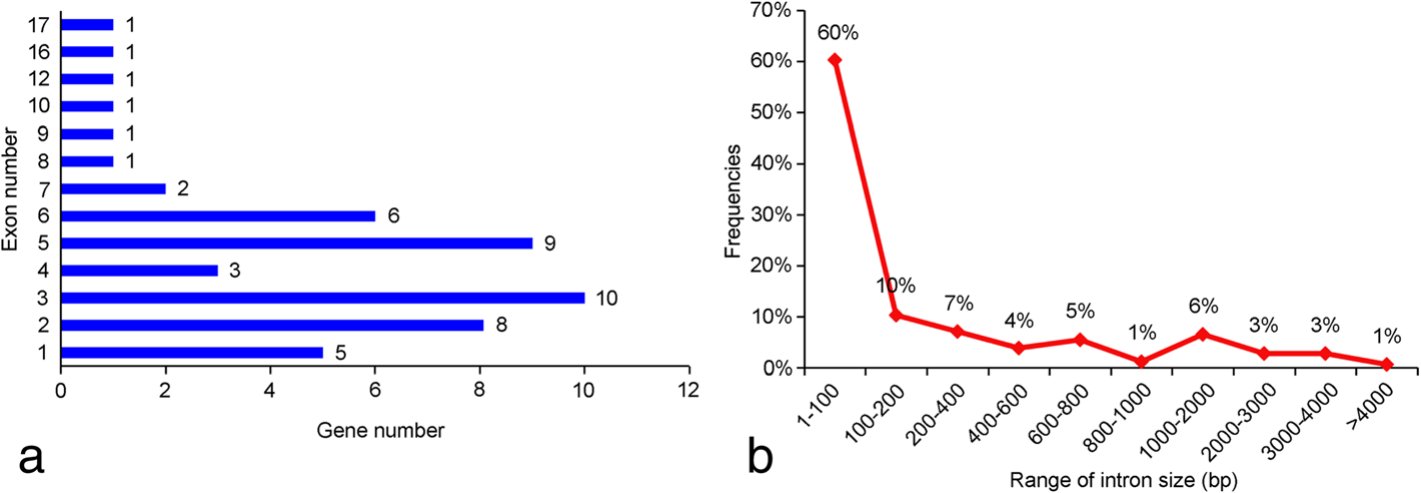
The number of exons (**a**) and the frequency of introns (**b**) of iGluRs in *Anopheles sinensis*

The 49 iGluR genes each have 1–17 exon, with 10 genes (20%) possessing 3 exons, followed by 9 (18%), 8 (16%) and 6 (12%) genes possessing 5, 2 and 6 exons, respectively (Additional file 3: Table S3, Fig. 1a). The 49 iGluR genes have a total of 186 introns with the lengths of these introns ranging from 17 bp to 4136 bp, and 1–100 bp introns have the most high frequencies 60% (112 introns in total), followed by 100–200 bp introns (19 introns, 10%), 200–400 bp (13, 7%), 1000–2000 bp (12, 6%) and 600–800 bp (10, 5%) (Additional file 3: Table S3, Fig. 1b). The phase0 (splicing between two codons), phase1 (between the first and second nucleotide of a codon) and phase2 (between the second and third nucleotide of a codon) [19] of introns account for 45.7% (85 in 186 introns), 24.7% (46) and 29.6% (55), respectively (Additional file 4: Figure S2).

**Fig. 2:**
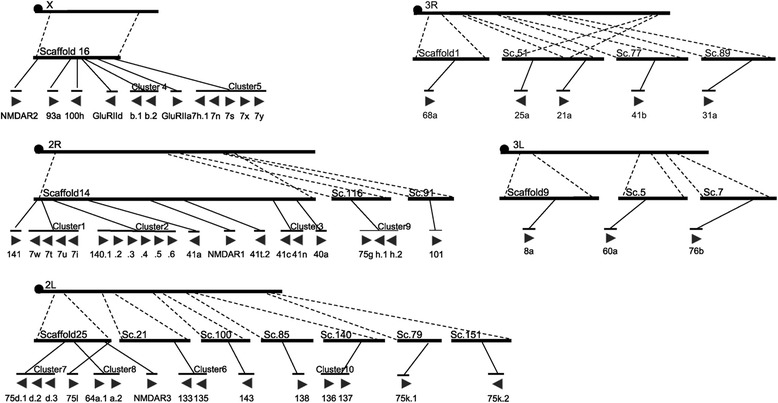
Scaffolds distribution and clusters of iGluR genes in *Anopheles sinensis* genome. The Scaffolds are syntenied to the chromosomes of *An. gambiae.* The filled triangles each represent one gene linked to the Scaffold mapped, with the gene name below the corresponding triangles and the direction of the regions indicating the direction of the 5′-3′ gene sequence. The gene Clusters recognized are marked with a transverse line above each cluster, which is connected to the same locus on the scaffold

The 56 iGluR genes were located on 18 scaffolds. These scaffolds were syntenied to 5 chromosomes, X, 2R, 2 L, 3R and 3 L in *An. gambiae* [22]. There were 17 genes (30.4% of 56), 12 (21.4%) and 7 (12.5%) to be distributed on Scaffold14, Scaffold16 and Scaffold25, respectively, and there were at most three genes on each of other scaffolds. The genes that located within a few thousand base pairs of each other and had similar function were considered to be a gene cluster [29]. A total of 10 clusters for 33 iGluR genes were mapped to 6 scaffolds (Scaffold14, 16, 21, 25, 116 and 140), and they were named as Cluster1–10 based on their distribution location (Fig. 2). Each cluster possessed 2–6 tandemly arranged genes that showed highly sequences similarity, and each cluster of genes might have the same origination and subsequent expansion via gene duplication events.

**Fig. 3:**
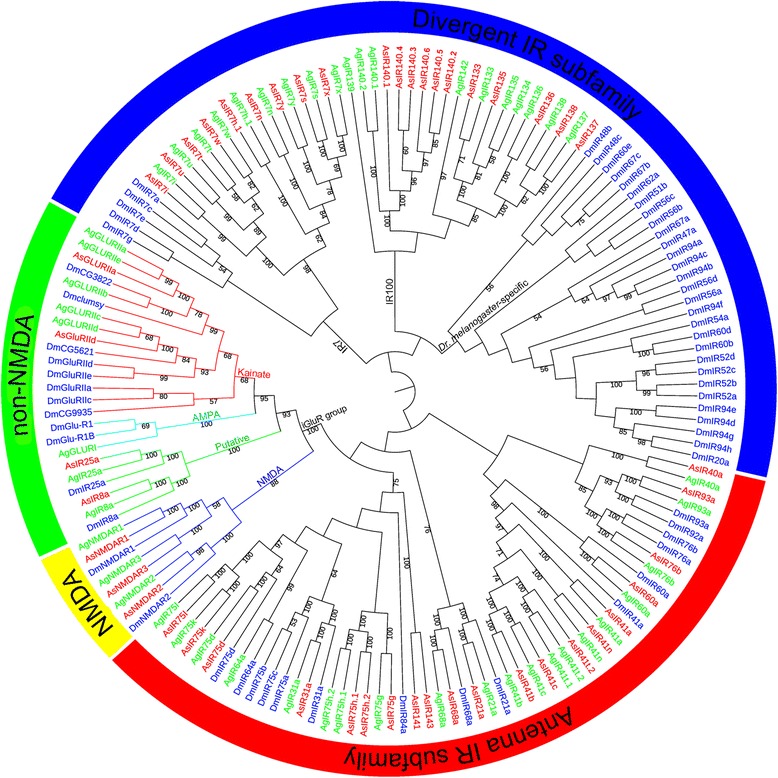
The domains, motifs and specific sites of iGluRs in *Anopheles sinensis*, *Anopheles gambiae* and *Drosophila melanogaster*. There are three domains, ATD (PF01094), LBD (PF10613) (containing S1 and S2), and Lig_Chan (PF00060), two motifs, Motif1 (part of transmembrane region M1) and Motif2 (part of transmembrane region M2). The specific sites for NMDA iGluRs: PBP2_iGluRs_NMDA with NMDA_Nr1/Nr2/Nr3 (cd13719/cd06378/cd13720) three types; the specific sites for non-NMDA iGluRs: PBP2_iGluRs_Kainate (cd13714) for Kainate, PBP2_iGluRs_AMPA (cd13715) for AMPA, and PBP2_iGluRs_Putative (cd13717) for Putative receptors

Several monophyletic clusters (see phylogenetic tree, Fig. 3) of IR genes separately existed in the same chromosome, suggesting an important role of intra-chromosomal translocation. For example, Cluster6 (AsIR133 and AsIR135) and Cluster10 (AsIR136 and AsIR137) tandemly arrayed on scaffold21 and scaffold140, respectively, and these two scaffolds both mapped to chromosome 2 L of *An. gambiae* (Fig. 2). This situation can also be found in *D. melanogaster*: 8 IR genes in IR94 orthologous groups located in three clusters, but separately and tandemly arrayed on chromosome 3R [7]. In addition, Cluster1 (AsIR7w, AsIR7t, AsIR7u and AsIR7i) and Cluster5 (AsIR7h.1, AsIR7n, AsIR7s, AsIR7x and AsIR7y) were in the same clade (phylogenetic tree, Fig. 3), and they were mapped to chromosome 2R and X of *An. gambiae*, respectively. Cluster7 (AsIR75d.1, AsIR75d.2 and AsIR75d.3) and Cluster9 (AsIR75g, AsIR75h.1 and AsIR75h.2) were in the same clade in the phylogenetic tree, and they were mapped to chromosome 2 L and 2R of *An. gambiae*, respectively. This situation indicated that non-allelic homology had also occurred frequently. Similar patterns were also observed in *D. melanogaster* and other drosophilid species [7]. Intra-chromosomal translocation and non-allelic homologous most likely occurred both during and after the formation of tandem arrays.

### Domains, motifs and specific sites of iGluRs

**Fig. 4:**
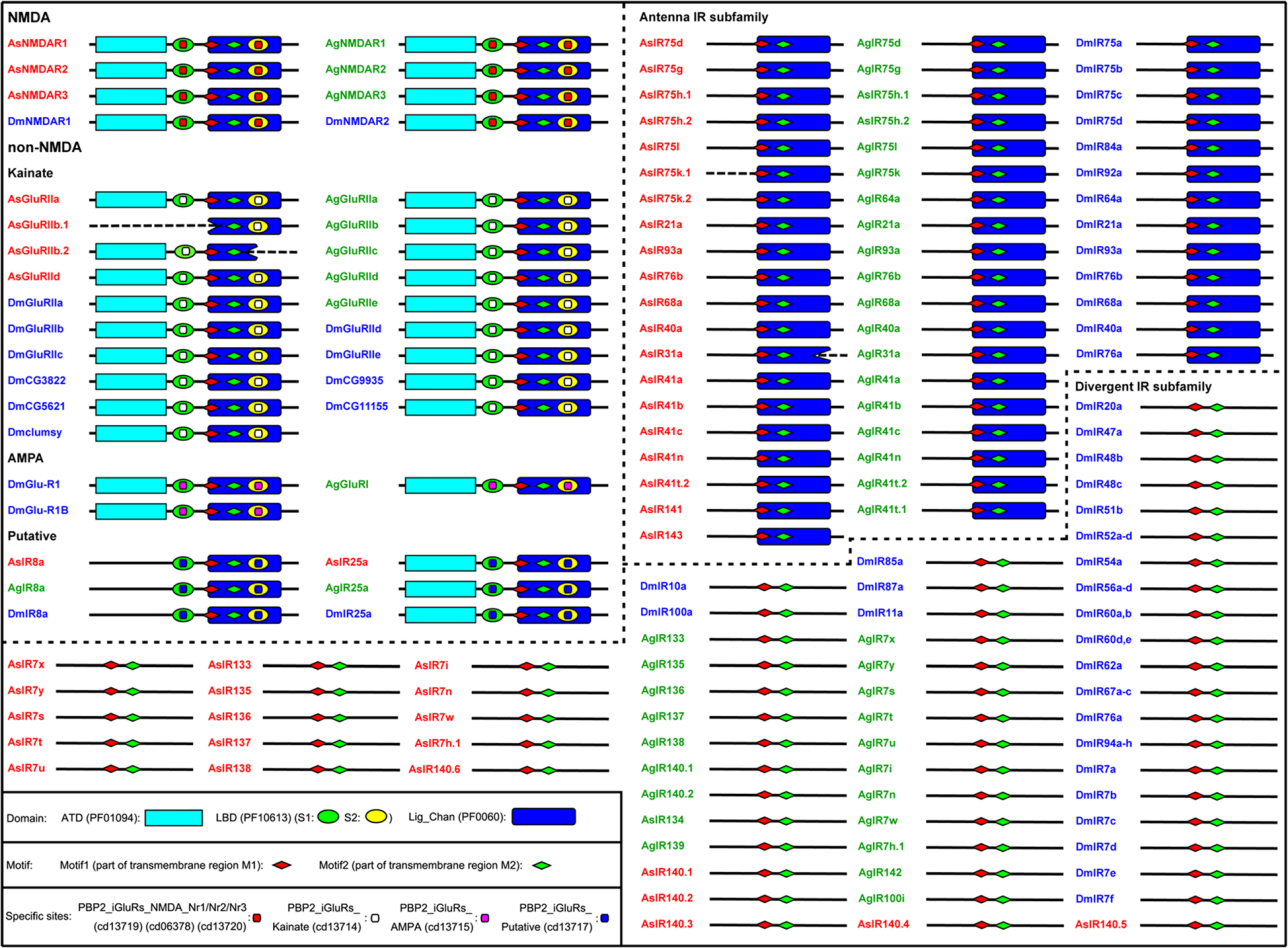
The Maximum Likelihood phylogenetic tree of iGluRs based on amino acid sequences in *Anopheles sinensis*, *Anopheles gambiae* and *Drosophila melanogaster*. The best evolutionary model WAG was selected and used in the phylogenetic analysis. The percent bootstrap values larger than 50% are marked on the nodes of the phylogenetic tree. *Abbreviations*: As, *An. sinensis*; Ag, *An. gambiae*; Dm, *Dm. melanogaster*. The names of amino acid sequences of *An. sinensis, An. gambiae* and *Dm. melanogaster* are marked in red, green and blue, respectively. NMDA, non-NMDA, Antenna IR subfamily and Divergent IR subfamily are indicated by yellow, green, red and blue arcs, respectively. The clades of NMDA, Kainate, AMPA and Putative in non-NMDA are depicted in blue, red, cyan and green, respectively

NMDA and non-NMDA class of aa sequences have three domains in *An. sinensis*, *An. gambiae* and *D. melanogaster*, the extracellular amino terminal domain (ATD), ligand binding domain (LBD), and Lig_Chan domain. The LBD contains two half-domains S1 and S2 that forms a ‘Venus flytrap’ structure and closes each other when LBD binds glutamate [30], and the Lig_Chan domain contains three transmembrane regions M1, M2, M3 and ion channel pore P [12]. The IR25a also possesses these three domains, and the IR8a has the LBD and Lig_Chan domain. However, other traditional IRs (i.e. traditional IRs but IR8a and IR25a) do not have any of these three domains with the exception of the Antenna IR subfamily that only has the Lig_Chan domain (Fig. 4, Additional file 5: Figure S1).

In the LBD domain, there are three conserved aa resides as glutamate ligand-binding sites. They are arginine (R) residue in S1 half-domain, which binds the glutamate α-carboxyl group, threonine (T) in the former half sequence of S2 half-domain, which contacts the glutamate γ-carboxyl group, and aspartate (D) or glutamate (E) in the latter half of the S2 half-domain, which interacts with the glutamate α-amino group [11]. All traditional iGluRs and IR8a in *An. sinensis*, *An. gambiae* and *D. melanogaster* have these three resides, and the IR25a only has R and D resides. However, these three resides in traditional IRs but IR8a and IR25a, especially in Divergent IR subfamily, were partially replaced by other aa, which suggests that these IRs might bind different ligands and conduct different functions. This result corresponds to the previous report that the LBD of IR family in *D. melanogaster* [11], *Cydia pomonella* [14], *Spodoptera littoralis* and *Bombyx mori* [31] were more variable and lack one or more ligand-binding sites that directly contact with glutamate ligand.

Two conserved motifs, Motif1 and Motif2 were identified in the present study for the first time in all iGluRs of *An. sinensis*, *An. gambiae* and *D. melanogaster* (Fig. 3, Additional file 5: Figure S1), and they are parts of M1 and M2 transmembrane region sequences in the Lig_Chan domain, respectively [12]. These two transmembrane regions M1 and M2 beside the ion channel pore P [32, 33]. The ion channel pore P is the characteristics of iGluRs as ligand gating ion channel receptors [11].

We also identified some specific sites in the LBD domain of traditional iGluRs, IR8a and IR25a in *An. sinensis*, *An. gambiae* and *D. melanogaster*. These sites contain two types of functional aa, Peptide binding [34] and Dimer interface aa. The Peptide binding aa are related to ligand glutamate binding; and the Dimer interface aa participate in the formation of dimer, which is necessary for receptor activation (phosphorylation) [35]. The specific sites for NMDA were PBP2_iGluRs_NMDA, which were divided into three types (NMDA_Nr1/Nr2/Nr3 (cd13719/cd06378/cd13720) based on the differences of their subunits’ component. The specific sites for Kainate and AMPA in non-NMDA were PBP2_iGluRs_Kainate (cd13714) and PBP2_iGluRs_AMPA (cd13715), respectively, and the specific sites for IR8a and IR25a were PBP2_iGluRs_Putative (cd13717). None of these specific sites were found in other traditional IRs (Fig. 4).

### Phylogenetics and classification of iGluRs

We constructed a phylogenetic tree based on all iGluRs in *An. sinensis*, *An. gambiae* and *D. melanogaster* using the ML method (Fig. 3). The result showed that these iGluRs could be divided into four groups, NMDA, non-NMDA, Antenna IR subfamily and Divergent IR subfamily in reference of previous classification [7, 14]. The Antenna IR subfamily was found to be paraphyletic, and the Divergent IR subfamily was resolved as an independent group but without high enough bootstrap support. The Divergent IR group was divided into three branches, IR7, IR100 and *D. melanogaster-*specific, which were all lacking high enough bootstrap support. This topology is largely consistent with the phylogenetic relationships established in *An. gambiae* and *D. melanogaster* [13]. Antenna IR genes were expressed in antennae, while Divergent IR of genes were expressed in gustatory organs or other tissues of insects [7, 14]. The *IR60a* of *An. gambiae* and *D. melanogaster* were classified to Divergent IR through expression and phylogenetic analysis [7, 11], and subsequently classified into Antenna IR through phylogenetic relationship [26]. Our results supported the latter, and the *IR60a* of *An. sinensis*, *An. gambiae* and *D. melanogaster* were classified to Antenna IR with a 100% bootstrap value.

The IR8a and IR25a, traditionally classified to Antenna IR [7], was shown to be monophyletic with a 100% of bootstrap support, and formed a clade with traditional non-NMDA as its sister group. Traditional non-NMDA contain two classes, Kainate and AMPA [36], and our study showed that these two classes both are monophyletic clades with 68% and 100% bootstrap support, respectively, which supported earlier classification. Traditional non-NMDA, IR8a and IR25a clustered together with a bootstrap value of 93%. This result is consistent with the previous studies on the phylogenetic relationships of iGluRs in *An. gambiae* and *D. melanogaster* [11, 26], *Ae. aegypti* and *Cx. quinquefasciatus* [7], and *C. pomonella* [14]. Based on the monophyly of IR8a and IR25a, and their close phylogenetic relationships and sequence features with traditional non-NMDA, we named the monophyletic group as Putative class and classified it to non-NMDA in the present study.

Our results also showed that NMDA is a monophyletic group with a bootstrap support of 88%, and it clustered with non-NMDA (Kainate, AMPA and Putative) as its sister clade. The NMDA and non-NMDA formed a new-defined monophyletic iGluR group (traditional iGluRs + Putative class) with a 100% bootstrap support. This result also corresponds to previous studies on iGluRs’ phylogenetic relationships for *C. pomonella* [14], *An. gambiae*, *D. melanogaster*, *Ae. aegypti* and *Cx. quinquefasciatus* [7].

*IR21a* and *IR25a* were earlier reported to express in antennae, and were required to mediate DOCC (Dorsal Organ Cool Cells) responses to cooling and for cool avoidance behavior both in *D. melanogaster* [37, 38] and *An. gambiae* [39]. These two genes have orthologues in *An. sinensis* (claded in the same branches with 100% bootstrap value of support), and they may also function associated with cooling and cool avoidance behavior.

*IR76b* was reported to express in both olfactory antennae and gustatory tissue proboscis in *D. melanogaster* [11], and it was characterized with highly conserved gustatory role in the detection of amino acids, in addition to its function as a salt taste receptor determined by genetic silencing and calcium imaging [40]. The *IR76b* orthologue also exists both in *An. gambiae* and *An. sinensis*, and may detect amino acids and respond to salt as well.

Three *Aedes albopictus* AalIR genes (*IR41a.2*, *IR87a.3* and *IR75d.2*) downregulated after a blood meal. Among them, both *IR41a.2* and *IR75d.2* have orthologues in *An. sinensis* and *An. gambiae* [41], and may also have a similar function in these two species of mosquitoes.

*IR84a* was reported to express in antennae of *D. melanogaster*, and was supposed as having a novel courtship function [39]. No homologue has been found in *An. sinensis* and *An. gambiae*, and *IR84a*-expressing neurons are activated by the aromatic odours phenylacetic acid and phenylacetaldehyde, which are widely found in food sources and oviposition sites for drosophilid flies. These findings reveal an effective evolutionary solution to coordinate feeding and oviposition site selection with reproductive behaviors through a specific sensory pathway.

### Comparison of different groups of iGluRs in *An. sinensis*

**Fig. 5:**
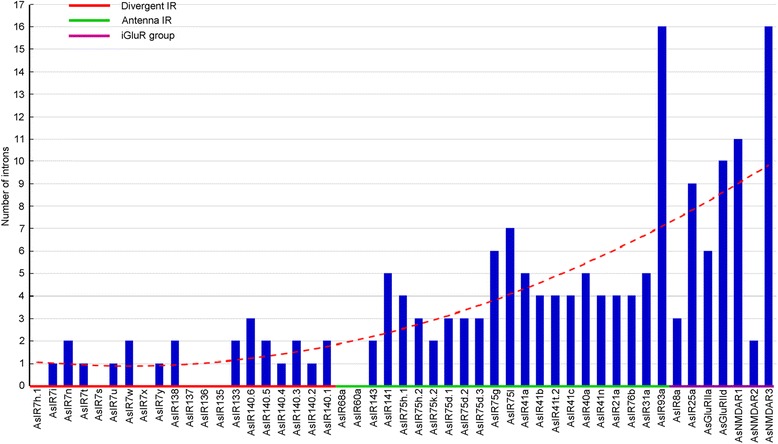
The changes of intron numbers of iGluR group, Antenna IR and Divergent IR in *An. sinensis*

**Fig. 6:**
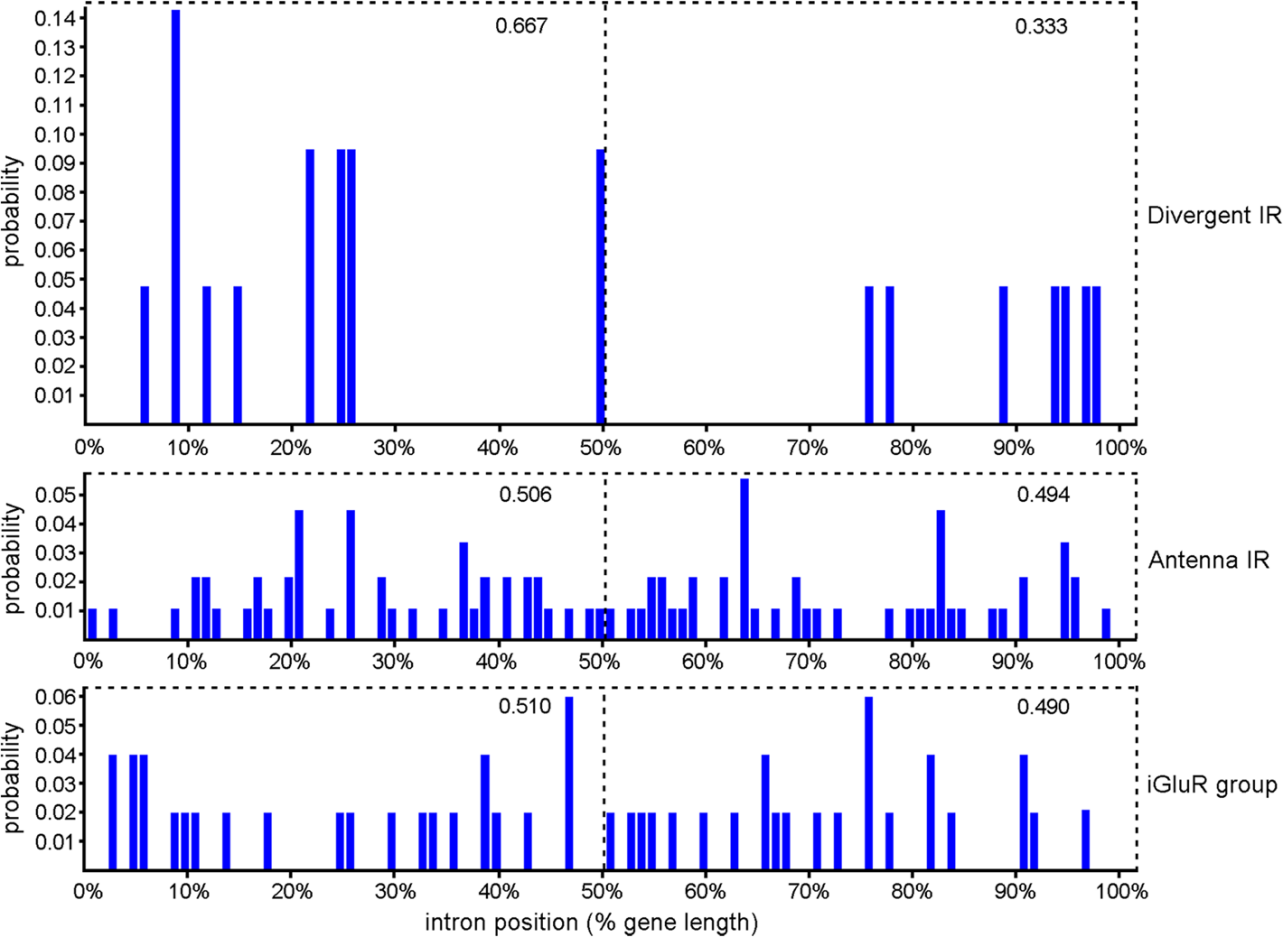
The distribution of intron positions as a percentage of gene length for iGluR group, Antenna IR and Divergent IR

We compared the gene structure and selection pressures among classification-updated iGluR group, Antenna IR and Divergent IR subfamily of genes in *An. sinensis* to understand their features. The intron numbers of iGluR group of genes range between 2 and 16 (average 8), while those of Antenna IR and Divergent IR ranged between 0 and 7 (average 4, except for 1 gene with 16 introns) and 0–3 (average 1), respectively (Fig. 5). This suggests that the gene structure of iGluR group is the most complicated, and in comparison that of the Divergent IR subfamily is the simplest. The frequencies of the introns’ occurrence locations in the iGluR group, Antenna IR and Divergent IR genes were 0.510 and 0.490, 0.506 and 0.494, and 0.667 and 0.333 in the former and latter half of protein-coding sequences (Fig. 6), respectively. This suggests that the introns of iGluR group and Antenna IR genes are averagely distributed on genes, but those of Divergent IR genes prefer the distribution on the first half (5′-end) of genes. This drastic intron loss phenomenon in multigene families is a hallmark of retroposition, which has been found in *An. gambia* and *D. melanogaster* [7] and may give rise to a new functional, intronless retrogene [42]. The reason for the observation that the introns of Divergent IR genes are more over the 5′-end might be the recombination of partially reverse-cDNA (a process which initiates at the 3′-end) with parental genes [43].

**Fig. 7:**
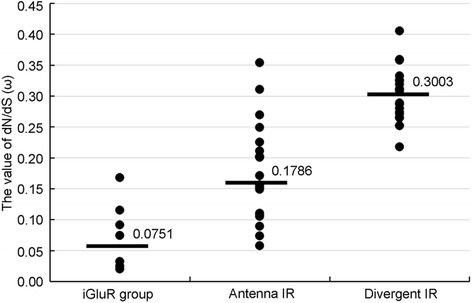
The comparison of the d_N_/d_S_ (ω) value of iGluR group, Antenna IR and Divergent IR in *An. sinensis*

The ratio (d_N_/d_S,_ ω) of nonsynonymous mutation (d_N_) and synonymous mutation (d_S_) can be used to judge whether selection pressures act on the protein-coding genes, as well as reflects their degree of conservation. The ω < 1 indicates that the gene is conducting purifying selection, ω = 1 indicates neutral selection, and ω > 1 indicates positive selection [44]. In this study, we calculated the ω values of the iGluR group, Antenna and Divergent IR subfamily of genes using corresponding *An. gambiae* iGluR sequences as reference. The results showed that the ω values for the iGluR group ranged between 0.0200–0.1679 (average 0.0751), and those for Antenna IR and Divergent IR subfamily ranged between 0.0577–0.3539 (average 0.1786) and 0.2519–0.4053 (average 0.3003), respectively (Fig. 7, Additional file 6: Table S4). These results suggest that the iGluR group of genes, with minimum ω values, evolve in strong purifying selection, which corresponds to their conservation in sequences and function of synaptic communication [5, 11]. The IR family of genes, especially the Divergent IR subfamily of genes, had a larger ω values, suggesting that they evolve under weaker purifying selection. The sequences of these genes are not so conserved, and might have more positive selected sites.

## Conclusions

In the study, a total of 56 iGluR genes were identified, named, and comprehensively analyzed in the whole genome of *An. sinensis*. These genes were located on 18 scaffolds, and 31 of them (29 being IRs) are distributed into 10 clusters that are suggested to result mainly from recent gene duplication. The iGluRs in *An. sinensis*, *An. gambiae* and *D. melanogaster* can be divided into four groups: NMDA, non-NMDA, Antenna IR and Divergent IR based on both characteristics comparison and phylogenetic analyses. The IR8a and IR25a were suggested to be monophiletic, named as Putative, and moved from traditional Antenna IR to traditional non-NMDA based on features and phylogenetic relationship. Traditional iGluRs and Putative together form a new concept of iGluR group, a group of genes that are relatively conserved and have smaller ω values and three ligand binding sites (but IR8a only has two). However, the IR family of genes, especially in the Divergent IR subfamily, were more variable with the larger ω values, and their ligand binding sites were highly variable. This study provides a comprehensive information framework for iGluRs, and is significant for further investigation of their functions.

## Additional files

Additional file 1: Table S1. Sequences information and basic biochemical characteristics of iGluRs in *Anopheles sinensis*. (XLS 69 kb)

Additional file 2: Table S2. The chromosome distribution of iGluRs in *Anopheles gambiae* and their comparison with *Anopheles sinensis* iGluRs. (DOCX 29 kb)

Additional file 3: Table S3. Detailed information of intron-exon organization of iGluR genes in *Anopheles sinensis*. (XLS 61 kb)

Additional file 4: Figure S2. Gene structure of iGluRs in *Anopheles sinensis. (TIFF 2527 kb)*

Additional file 5: Figure S1. The domains, motifs and specific sites annotation of iGluR group (a), Antenna IR subfamily (b) and Divergent IR subfamily (c) in *An. sinensis*, *An. gambiae* and *D. melanogaster* through multiple alignment. The amino terminal domain (ATD) is represented with “ATD” in red. The ligand binding domain (LBD) is marked with black lines above, which is consisted with S1 and S2 two half-domains. And the Lig_Chan domain is marked with blue lines above, which is consists of three transmembrane regions M1, M2, M3 and an ion channel pore (P). The black-lined boxes are ligand-gated sites. The functional aa of Peptides binding and Dimer interface of specific sites have a background of yellow and green, respectively. The amino acids in lows with 100%, 70–99% and below 70% identity are denoted with black, grey and white shade, respectively. *Abbreviations*: As, *An. sinensis*; Ag, *An. gambiae*; Dm, *D. melanogaster. (PDF 7939 kb)*

Additional file 6: Table S4. The value of d_N_, d_S_ and d_N_/d_S_(ω) of iGluR genes in *Anopheles sinensis. (DOCX 21 kb)*

Additional file 7: Table S5. The accession numbers of iGluRs in *An. gambiae* and *D. melanogaster. (XLS 31 kb)*

## Abbreviations

Aa: Amino acid
ATD: Amino terminal domain
iGluRs: Ionotropic glutamate receptors
IRs: Ionotropic receptors
LBD: Ligand binding domain
ML: Maximum likelihood method
