# Supplementary material for: Genome-wide identification, characterization and classification of ionotropic glutamate receptor genes (iGluRs) in the malaria vector Anopheles sinensis (Diptera: Culicidae)
Source: Parasit Vectors. 2018 Jan 15;11:34. doi: 10.1186/s13071-017-2610-x (PMC5769321; doi:10.1186/s13071-017-2610-x)
Supplement: Supplementary file 2 — The chromosome distribution of iGluRs in Anopheles gambiae and their comparison with Anopheles sinensis iGluRs. (DOCX 29 kb) [file 13071_2017_2610_MOESM2_ESM.docx]

TableS2 The chromosome distribution of iGluRs in *Anopheles gambiae* and their comparison with *Anopheles sinensis* iGluRs

| ***An. sinensis*** | | ***An. gambiae*** | **Vectorbase ID(Ag)** | | **Chromosome distribution** | **Blast condition** | | | | | |
| --- | --- | --- | --- | --- | --- | --- | --- | --- | --- | --- | --- |
|  |  |  |  |  |  | **pep. length(a.a.)** | | | | | **similarity** |
|  |  |  |  |  |  | **As** | **Ag** | | | |  |
| **NMDA** | | | | | | | | | | | |
| *AsNMDAR1* | | *AgNMDAR1* | AGAP001478 | | 2R: 5,390,305-5,394,755 | 963 | 966 | | | 0.97 | |
| *AsNMDAR2* | | *AgNMDAR2* | AGAP012429 | | unk: 284,129-400,942 | 930 | 935 | | | 0.95 | |
| *AsNMDAR3* | | *AgNMDAR3* | AGAP005527 | | 2L: 16,640,867-16,647,151 | 1788 | 1762 | | | 0.74 | |
| **Non-NMDA: AMPA** | | | | | | | | | | | |
| *-* | | *AgGLURI* | AGAP006027 | | 2L: 25,274,226-25,314,781 | - | - | | | - | |
| **Non-NMDA: Kainate** | | | | | | | | | | | |
| *AsGluRIIa* | *AgGLURIIa* | | | AGAP000803 | X: 14,739,086-14,744,472 | 939 | | 939 | 0.89 | | |
| *AsGluRIIb.1* | *AgGLURIIb* | | | AGAP000801 | X: 14,618,285-14,702,072 | 362 | | 888 | 0.73 | | |
| *AsGluRIIb.2* |  |  |  |  |  | 632 | |  | 0.91 | | |
| *-* | *AgGLURIIc* | | | AGAP000798 | X: 14,600,998-14,612,301 | - | | - | - | | |
| *AsGluRIId* | *AgGLURIId* | | | AGAP002797 | 2R: 27,638,116-27,644,376 | 1038 | | 978 | 0.93 | | |
| *-* | *AgGLURIIe* | | | AGAP012447 | unk: 1,553,896-1,557,262 | - | | - | - | | |
| **Non-NMDA: Putative** | | | | | | | | | | | |
| *AsIR8a* | *AgIR8a* | | | AGAP010411 | 3L: 2,867,389-2,870,222 | 1153 | | 828 | 0.76 | | |
| *AsIR25a* | *AgIR25a* | | | AGAP010272 | 3R: 51,917,004-51,920,704 | 946 | | 914 | 0.97 | | |
| **IR: Antenna IR subfamily** | | | | | | | | | | | |
| *AsIR21a* | *AgIR21a* | | | AGAP008511 | 3R: 11,468,382-11,471,590 | 957 | | 466 | 0.74 | | |
| *AsIR31a* | *AgIR31a* | | | AGAP009014 | 3R: 23,550,606-23,552,617 | 586 | | 523 | 0.76 | | |
| *AsIR75d.1* | *AgIR75d* | | | AGAP004969 | 2L: 7,301,252-7,303,528 | 645 | | 645 | 0.72 | | |
| *AsIR75d.2* |  |  |  |  |  | 645 | |  | 0.72 | | |
| *AsIR75d.3* |  |  |  |  |  | 645 | |  | 0.72 | | |
| *AsIR75g* | *AgIR75g* | | | AGAP013085 | 2R: 10,846,334-10,848,911 | 387 | | 287 | 0.48 | | |
| *AsIR75h.1* | *AgIR75h.1* | | | AGAP001811 | 2R: 10,851,283-10,853,577 | 593 | | 647 | 0.52 | | |
| *AsIR75h.2* | *AgIR75h.2* | | | AGAP001812 | 2R: 10,855,568-10,857,858 | 602 | | 647 | 0.49 | | |
| *AsIR75k.1* | *AgIR75k* | | | AGAP007498 | 2L: 46,923,249-46,925,420 | 647 | | 675 | 0.71 | | |
| *AsIR75k.2* |  |  |  |  |  | 430 | | 675 | 0.81 | | |
| *AsIR75l* | *AgIR75l* | | | AGAP005466 | 2L: 15,949,821-15,952,169 | 650 | | 641 | 0.70 | | |
| *AsIR60a* | *AgIR60a* | | | AGAP011943 | 3L: 35,436,382-35,438,481 | 704 | | 646 | 0.75 | | |
| *AsIR64a.1* | *AgIR64a* | | | AGAP004923 | 2L: 6,087,139-6,107,636 | 647 | | 567 | 0.73 | | |
| *AsIR64a.2* |  |  |  |  |  | 440 | |  | 0.61 | | |
| *AsIR68a* | *AgIR68a* | | | AGAP007951 | 3R: 3,275,939-3,278,326 | 773 | | 625 | 0.61 | | |
| *AsIR40a* | *AgIR40a* | | | AGAP004021 | 2R: 47,922,057-47,924,755 | 753 | | 742 | 0.84 | | |
| *AsIR41a* | *AgIR41a* | | | AGAP002904 | 2R: 29,130,386-29,133,257 | 646 | | 637 | 0.66 | | |
| *AsIR41b* | *AgIR41b* | | | AGAP008759 | 3R: 17,137,047-17,139,240 | 658 | | 391 | 0.66 | | |
| *AsIR41c* | *AgIR41c* | | | AGAP012951 | 2R: 39,202,960-39,205,188 | 561 | | 663 | 0.58 | | |
| *AsIR41n* | *AgIR41n* | | | AGAP003531 | 2R: 39,207,749-39,210,030 | 644 | | 635 | 0.61 | | |
| *AsIR41t.2* | *AgIR41t.1* | | | AGAP004432 | 2R: 55,960,058-55,962,321 | - | | - | - | | |
|  | *AgIR41t.2* | | | AGAP012969 | 2R: 55,988,209-55,990,472 | 644 | | 627 | 0.55 | | |
| *AsIR76b* | *AgIR76b* | | | AGAP011968 | 3L: 35,687,053-35,692,621 | 613 | | 474 | 0.85 | | |
| *AsIR93a* | *AgIR93a* | | | AGAP000256 | X: 4,900,441-4,905,540 | 825 | | 857 | 0.78 | | |
| *AsIR143* | *-* | | | - | - | - | | - | - | | |
| *AsIR141* | *AgIR141* | | | AGAP013473 | 2R: 31,458,247-31,463,898 | 406 | | 307 | 0.41 | | |
| **IR: Divergent IR subfamily** | | | | | | | | | | | |
| *-* | *AgIR100a* | | | AGAP000140 | X: 2,149,577-2,152,578 | - | | - | - | | |
| *AgIR100h* | *AgIR100h* | | | AGAP000293 | X: 5,199,638-5,204,600 | 535 | | 700 | 0.44 | | |
| *-* | *AgIR100i* | | | AGAP004475 | 2R: 56,749,587-56,751,832 | - | | - | - | | |
| *AgIR101* | *AgIR101* | | | AGAP013425 | 2R: 42,015,346-42,017,474 | 671 | | 673 | 0.69 | | |
| *AgIR133* | *AgIR133* | | | AGAP005677 | 2L: 18,651,360-18,653,143 | 536 | | 551 | 0.40 | | |
| *-* | *AgIR134* | | | AGAP005678 | 2L: 18,657,551-18,659,242 | - | | - | - | | |
| *AgIR135* | *AgIR135* | | | AGAP005679 | 2L: 18,662,281-18,664,071 | 561 | | 572 | 0.49 | | |
| *AgIR136* | *AgIR136* | | | AGAP006440 | 2L: 31,959,226-31,960,962 | 333 | | 578 |  | | |
| *AgIR137* | *AgIR137* | | | AGAP006440 | 2L: 31,957,279-31,958,959 | 397 | | 559 |  | | |
| *AgIR138* | *AgIR138* | | | AGAP006440 | 2L: 31,954,985-31,956,676 | 913 | | 1074 | 0.45 | | |
| *-* | *AgIR139* | | | AGAP006691 | 2L: 36,737,099-36,738,805 | - | | - | - | | |
| *As140.1* | *AgIR140.1* | | | AGAP013242 | 2R: 16,645,150-16,647,020 | 584 | | 600 | 0.51 / 0.49 | | |
| *As140.2* |  |  |  |  |  | 543 | |  | 0.49 / 0.51 | | |
| *As140.3* |  |  |  |  |  | 526 | |  | 0.49 / 0.48 | | |
| *As140.4* | *AgIR140.2* | | | AGAP013436 | 2R: 16,642,268-16,643,951 | 360 | | 527 | 0.51 / 0.51 | | |
| *As140.5* |  |  |  |  |  | 585 | |  | 0.48 / 0.50 | | |
| *As140.6* |  |  |  |  |  | 511 | |  | 0.47 / 0.47 | | |
| *-* | *AgIR142* | | | AGAP006407 | 2L: 31,561,183-31,564,566 | - | | - | - | | |
| *AsIR7h.1* | *AgIR7h.1* | | | AGAP013154 | X: 12,751,181-12,753,181 | 622 | | 666 | 0.52 | | |
| *AsIR7i* | *AgIR7i* | | | AGAP013363 | 2R: 26,793,376-26,795,314 | 612 | | 620 | 0.49 | | |
| *AsIR7n* | *AgIR7n* | | | AGAP000714 | X: 12,757,892-12,760,135 | 606 | | 747 | 0.47 | | |
| *AsIR7s* | *AgIR7s* | | | AGAP013409 | X: 12,762,561-12,764,534 | 651 | | 657 | 0.59 | | |
| *AsIR7t* | *AgIR7t* | | | AGAP002763 | 2R: 26,797,736-26,799,713 | 623 | | 634 | 0.50 | | |
| *AsIR7u* | *AgIR7u* | | | AGAP013285 | 2R: 26,795,525-26,797,440 | 584 | | 612 | 0.56 | | |
| *AsIR7w* | *AgIR7w* | | | AGAP013416 | 2R: 26,799,941-26,801,942 | 636 | | 645 | 0.53 | | |
| *AsIR7x* | *AgIR7x* | | | AGAP013520 | X: 12,767,186-12,769,138 | 619 | | 650 | 0.55 | | |
| *AsIR7y* | *AgIR7y* | | | AGAP013172 | X: 12,771,474-12,773,520 | 661 | | 652 | 0.61 | | |
