# Supplementary figures and images for: Genome-wide identification, characterization and classification of ionotropic glutamate receptor genes (iGluRs) in the malaria vector Anopheles sinensis (Diptera: Culicidae)
Source: Parasit Vectors. 2018 Jan 15;11:34. doi: 10.1186/s13071-017-2610-x (PMC5769321; doi:10.1186/s13071-017-2610-x)

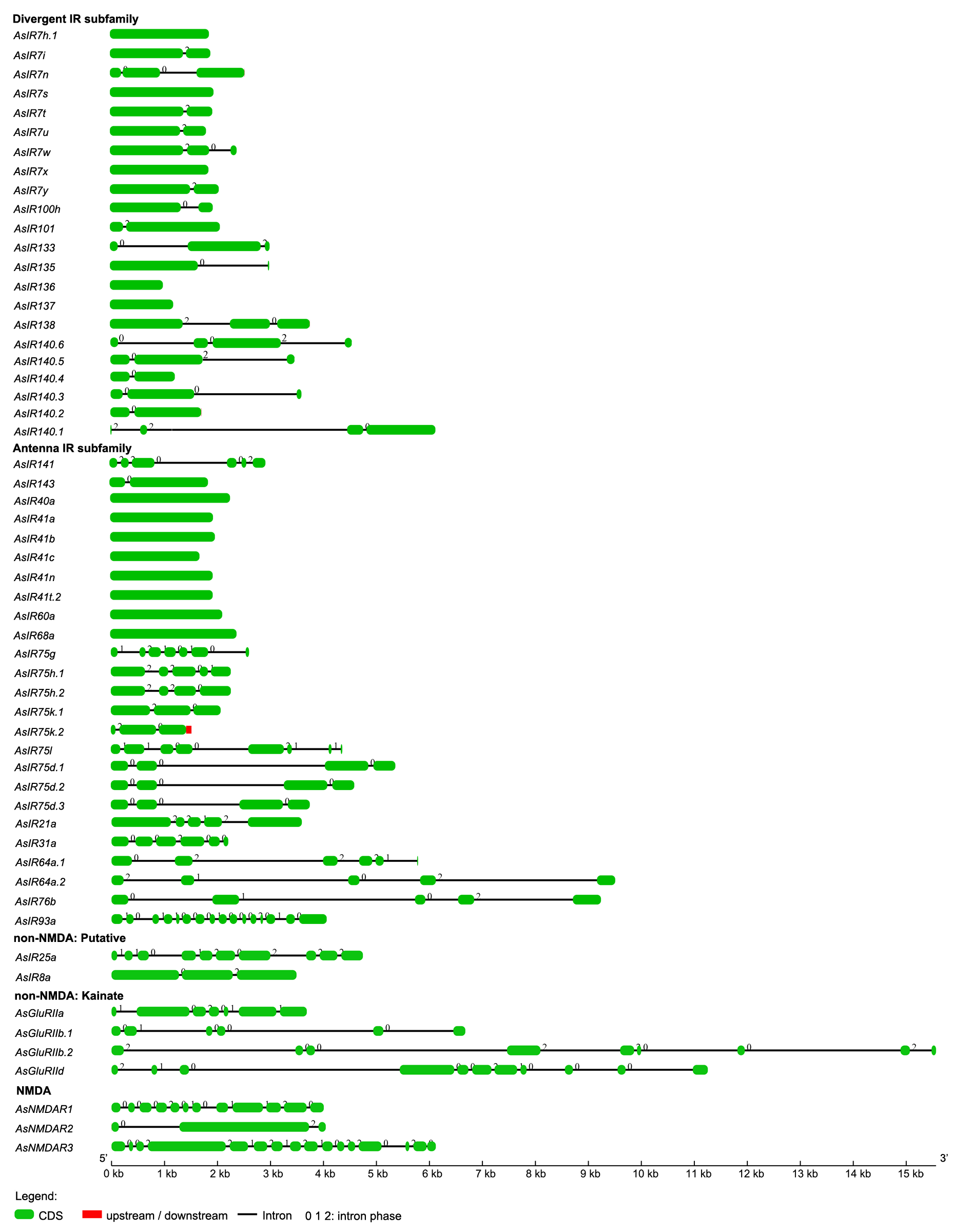

Supplement: Supplementary file 4 — Gene structure of iGluRs in Anopheles sinensis. (TIFF 2527 kb) [file 13071_2017_2610_MOESM4_ESM.tif]
