# Supplementary material for: Genome-wide identification, characterization and classification of ionotropic glutamate receptor genes (iGluRs) in the malaria vector Anopheles sinensis (Diptera: Culicidae)
Source: Parasit Vectors. 2018 Jan 15;11:34. doi: 10.1186/s13071-017-2610-x (PMC5769321; doi:10.1186/s13071-017-2610-x)
Supplement: Supplementary file 5 — The domains, motifs and specific sites annotation of iGluR group (a), Antenna IR subfamily (b) and Divergent IR subfamily (c) in An. sinensis, An. gambiae and D. melanogaster through multiple alignment. The amino terminal domain (ATD) is represented with “ATD” in red. The ligand binding domain (LBD) is marked with black lines above, which is consisted with S1 and S2 two half-domains. And the Lig_Chan domain is marked with blue lines above, which is consists of three transmembrane regions M1, M2, M3 and an ion channel pore (P). The black-lined boxes are ligand-gated sites. The functional aa of Peptides binding and Dimer interface of specific sites have a background of yellow and green, respectively. The amino acids in lows with 100%, 70–99% and below 70% identity are denoted with black, grey and white shade, respectively. Abbreviations: As, An. sinensis; Ag, An. gambiae; Dm, D. melanogaster. (PDF 7939 kb) [file 13071_2017_2610_MOESM5_ESM.pdf]

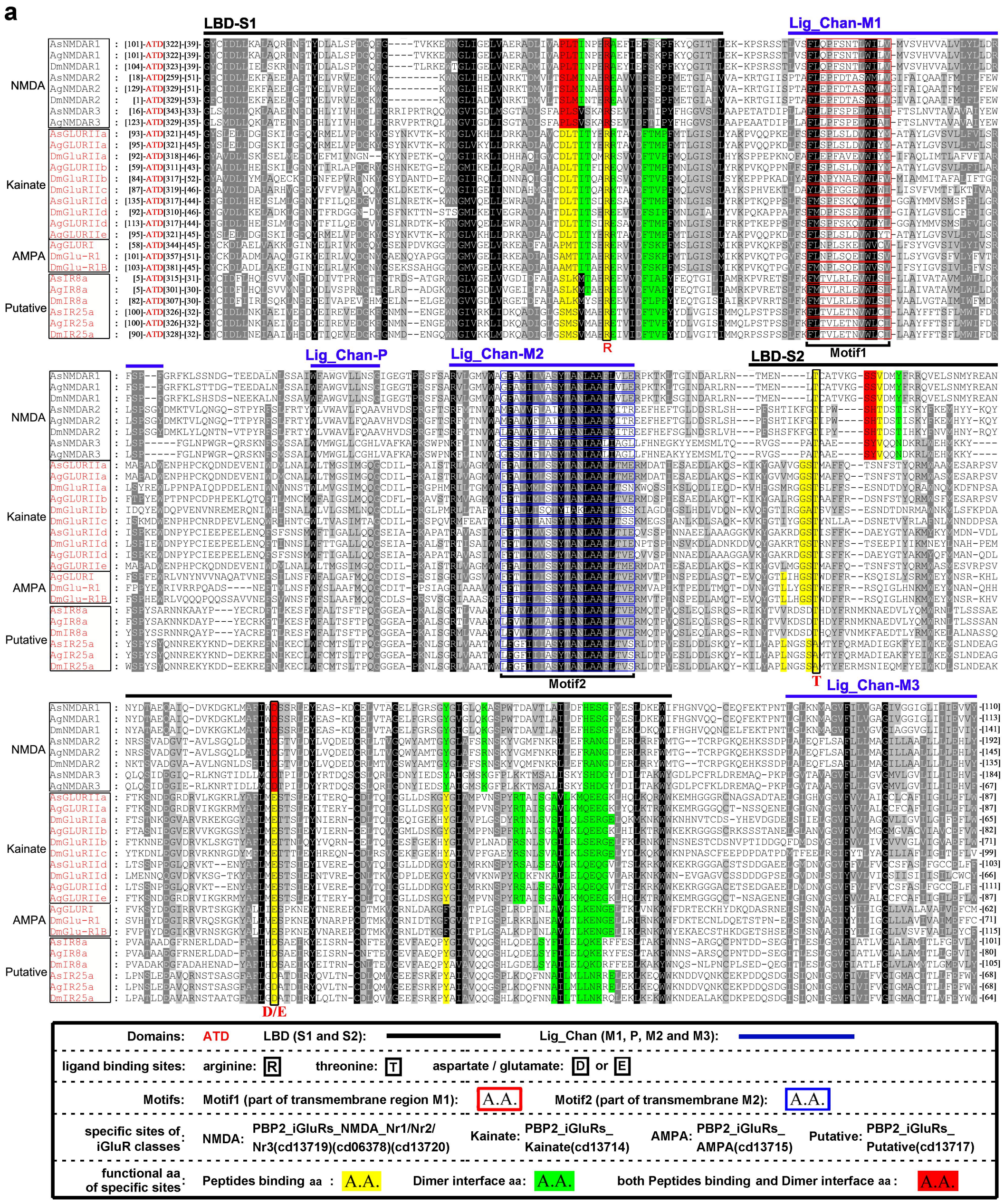
a
LBD-S1
Lig_Chan-M1
Lig_Chan-P
Lig_Chan-M2
LBD-S2
Lig_Chan-M3
Motif1
Motif2
NMDA
Kainate
AMPA
Putative
R
T
D/E
Domains: ATD LBD (S1 and S2): Lig_Chan (M1, P, M2 and M3):
ligand binding sites: arginine: R threonine: T aspartate / glutamate: D or E
Motifs: Motif1 (part of transmembrane region M1): A.A. Motif2 (part of transmembrane M2): A.A.
specific sites of iGluR classes: NMDA: PBP2_iGluRs_NMDA_Nr1/Nr2/ Nr3(cd13719)(cd06378)(cd13720) Kainate: PBP2_iGluRs_ Kainate(cd13714) AMPA: PBP2_iGluRs_ AMPA(cd13715) Putative: PBP2_iGluRs_ Putative(cd13717)
functional aa of specific sites: Peptides binding aa : A.A. Dimer interface aa: A.A. both Peptides binding and Dimer interface aa : A.A.

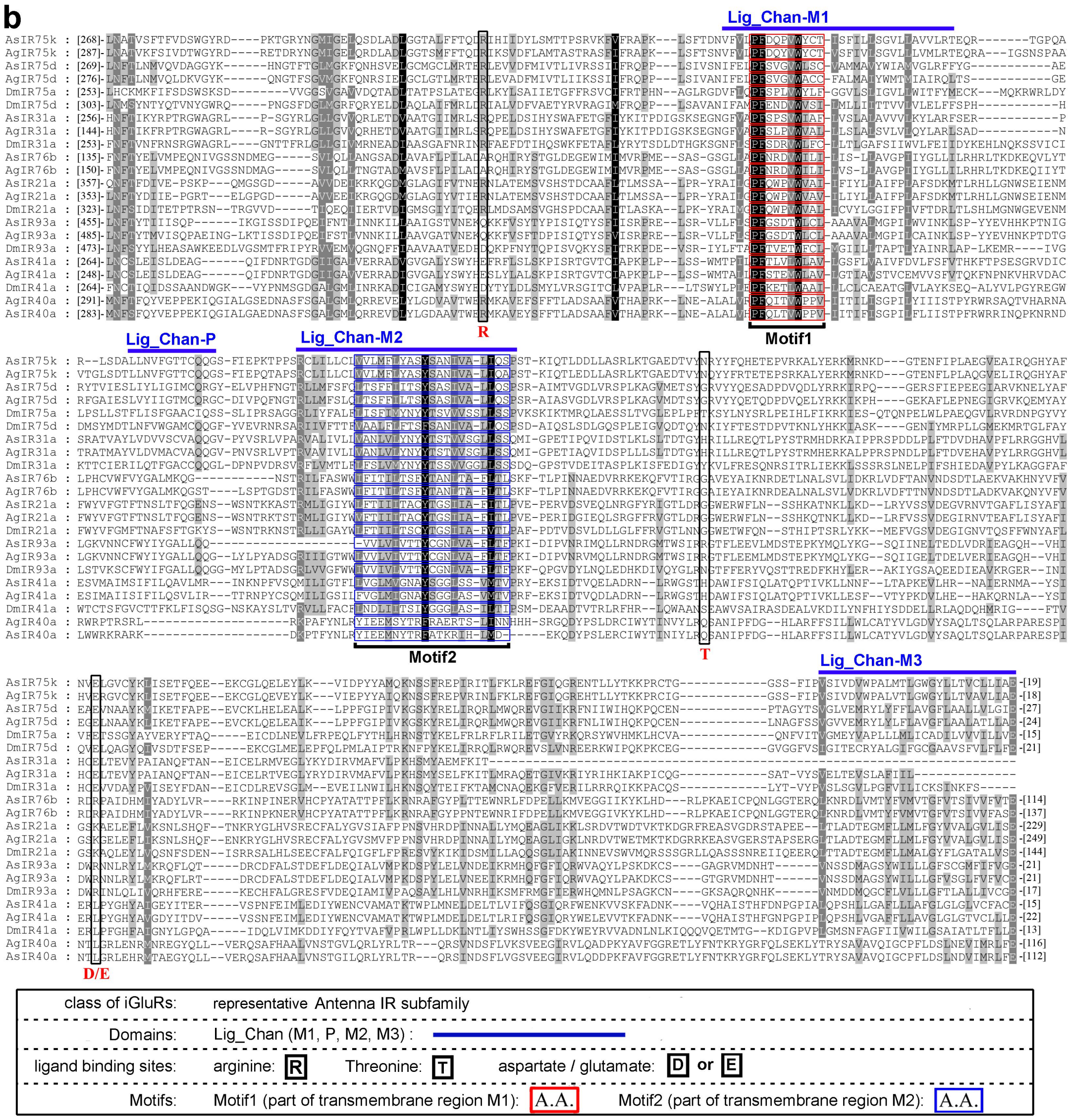
b
Lig_Chan-M1
Motif1
R
Lig_Chan-P
Lig_Chan-M2
Motif2
T
Lig_Chan-M3
D/E
class of iGluRs: representative Antenna IR subfamily
Domains: Lig_Chan (M1, P, M2, M3) :
ligand binding sites: arginine: R Threonine: T aspartate / glutamate: D or E
Motifs: Motif1 (part of transmembrane region M1): A.A. Motif2 (part of transmembrane region M2): A.A.

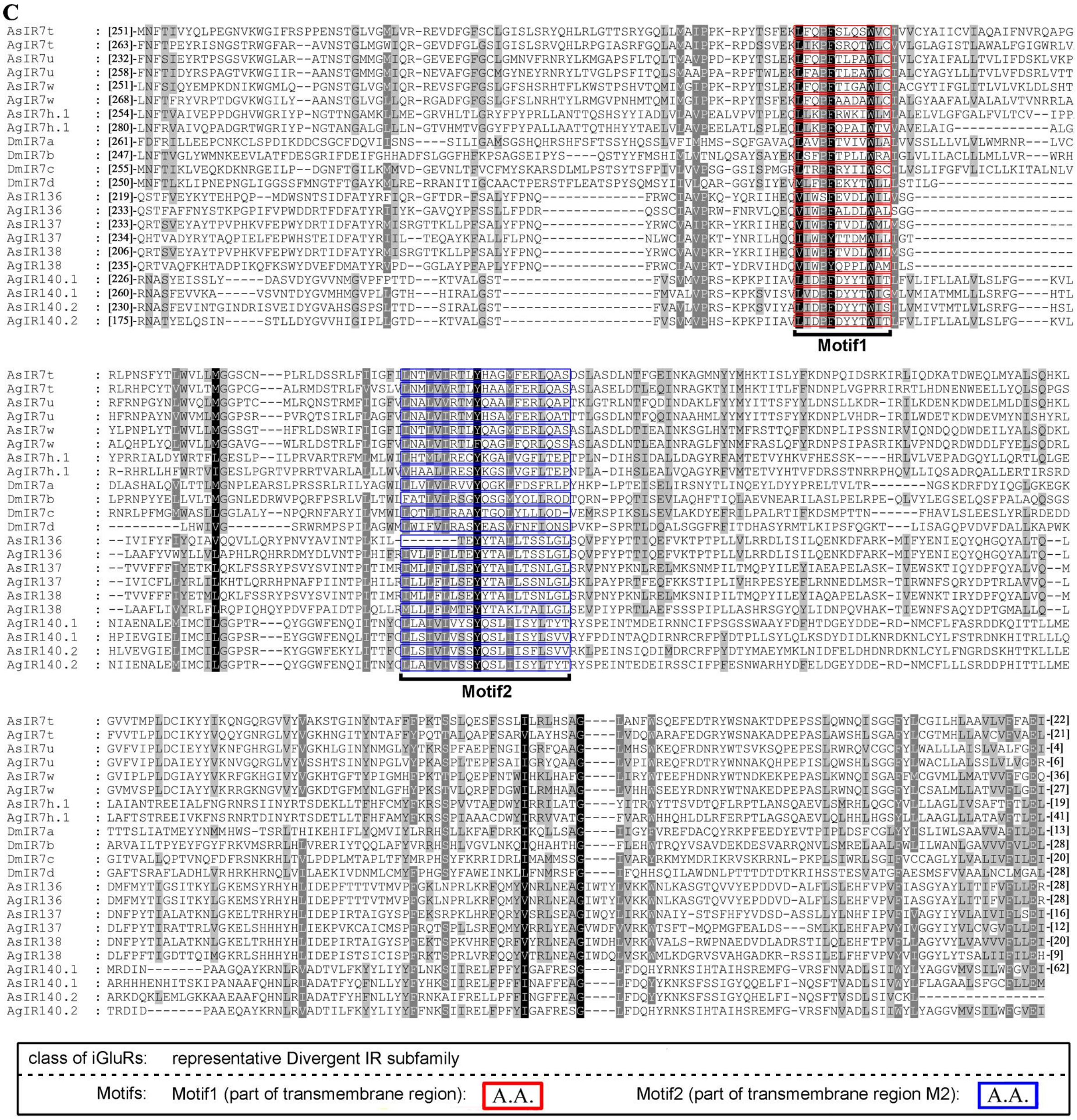
C
AsIR7t
AgIR7t
AsIR7u
AgIR7u
AsIR7w
AgIR7w
AsIR7h.1
AgIR7h.1
DmIR7a
DmIR7b
DmIR7c
DmIR7d
AsIR136
AgIR136
AsIR137
AgIR137
AsIR138
AgIR138
AgIR140.1
AsIR140.1
AsIR140.2
AgIR140.2
Motif1
Motif2
class of iGluRs: representative Divergent IR subfamily
Motifs: Motif1 (part of transmembrane region): A.A.
Motif2 (part of transmembrane region M2): A.A.
