# Supplementary material for: Genome-wide identification, characterization and classification of ionotropic glutamate receptor genes (iGluRs) in the malaria vector Anopheles sinensis (Diptera: Culicidae)
Source: Parasit Vectors. 2018 Jan 15;11:34. doi: 10.1186/s13071-017-2610-x (PMC5769321; doi:10.1186/s13071-017-2610-x)
Supplement: Supplementary file 6 — The value of dN, dS and dN/dS(ω) of iGluR genes in Anopheles sinensis. (DOCX 21 kb) [file 13071_2017_2610_MOESM6_ESM.docx]

**Table S4** The value of d_N_, d_S_ and d_N_/d_S_ (ω) of iGluR genes in *Anopheles sinensis*

| **iGluRs** | **d_N_** | **d_S_** | d_N_/d_S_ (**ω)** | **Average** |
| --- | --- | --- | --- | --- |
| **NMDA** | | | | |
| *AsNMDAR1* | 0.0232 | 0.9388 | 0.0247 |  |
| *AsNMDAR2* | 0.0780 | 0.2430 | 0.0321 |  |
| *AsNMDAR3* | 0.1749 | 1.0418 | 0.1679 |  |
| **non-NMDA: Kainate** | | | | |
| *AsGluRIIa* | 0.0678 | 0.7417 | 0.0914 |  |
| *AsGluRIId* | 0.0581 | 0.7812 | 0.0743 |  |
| **non-NMDA: Putative** | | | | |
| *AsIR8a* | 0.1628 | 1.4129 | 0.1152 |  |
| *AsIR25a* | 0.0403 | 2.0132 | 0.0200 | 0.0751 |
| **IR: Antenna IR subfamily** | | | | |
| *AgIR60a* | 0.1723 | 0.8587 | 0.2007 |  |
| *AgIR141* | 0.5778 | 1.8605 | 0.3106 |  |
| *AsIR21a* | 0.1163 | 1.3034 | 0.0892 |  |
| *AsIR31a* | 0.1069 | 0.9931 | 0.1076 |  |
| *AsIR40a* | 0.1283 | 1.7438 | 0.0736 |  |
| *AsIR41a* | 0.2442 | 1.0837 | 0.2253 |  |
| *AsIR41b* | 0.2720 | 1.7416 | 0.1562 |  |
| *AsIR41c* | 0.4256 | 1.5797 | 0.2694 |  |
| *AsIR41n* | 0.2922 | 1.3850 | 0.2110 |  |
| *AsIR41t.2* | 0.2984 | 2.0039 | 0.1489 |  |
| *AsIR68a* | 0.2127 | 0.8538 | 0.2491 |  |
| *AsIR75d.1* | 0.1950 | 0.9665 | 0.2018 |  |
| *AsIR75d.2* | 0.1950 | 0.9665 | 0.2018 |  |
| *AsIR75d.3* | 0.1950 | 0.9665 | 0.2018 |  |
| *AsIR75g* | 0.6808 | 1.9237 | 0.3539 |  |
| *AsIR75h.1* | 0.3454 | 2.2373 | 0.1544 |  |
| *AsIR75h.2* | 0.3737 | 2.4707 | 0.1513 |  |
| *AsIR75I* | 0.2233 | 1.3054 | 0.1711 |  |
| *AsIR75k.2* | 0.1282 | 1.1627 | 0.1102 |  |
| *AsIR76b* | 0.0456 | 0.7896 | 0.0577 |  |
| *AsIR93a* | 0.111 | 1.0564 | 0.1051 | 0.1786 |
| **IR: Divergent IR subfamily** | | | | |
| *AsIR7x* | 0.3804 | 1.4346 | 0.2651 |  |
| *AsIR7y* | 0.3062 | 1.2148 | 0.2521 |  |
| *AsIR7s* | 0.3466 | 1.1253 | 0.3080 |  |
| *AsIR7t* | 0.4532 | 1.2665 | 0.3579 |  |
| *AsIR7u* | 0.3722 | 1.1424 | 0.3258 |  |
| *AsIR7i* | 0.4913 | 1.7022 | 0.2887 |  |
| *AsIR7n* | 0.4732 | 1.2176 | 0.3591 |  |
| *AsIR7w* | 0.3978 | 1.8271 | 0.2177 |  |
| *AsIR7h.1* | 0.3978 | 0.9817 | 0.4053 |  |
| *AsIR133* | 0.6027 | 2.3926 | 0.2519 |  |
| *AsIR135* | 0.4355 | 1.606 | 0.2712 |  |
| *AsIR136* | 0.5698 | 1.7551 | 0.3247 |  |
| *AsIR137* | 0.4754 | 1.6571 | 0.2869 |  |
| *AsIR138* | 0.4877 | 1.7821 | 0.2737 |  |
| *AsIR140.1* | 0.4443 | 1.4181 | 0.3113 |  |
| *AsIR140.2* | 0.4522 | 1.6155 | 0.2799 |  |
| *AsIR140.3* | 0.4893 | 1.8486 | 0.2647 |  |
| *AsIR140.4* | 0.4459 | 1.3410 | 0.3325 |  |
| *AsIR140.5* | 0.4732 | 1.5238 | 0.3105 |  |
| *AsIR140.6* | 0.4767 | 1.4950 | 0.3189 | 0.3003 |

d_N_: nonsynonymous, d_S_: synonymous. The genes in this table all have complete amino acid sequences and have orthologous genes in *An. gambiae*.
